# Developing a Concept on Ethical, Legal and Social Implications (ELSI) for Data Literacy in Health Professions: A Learning Objective-Based Approach

**DOI:** 10.3390/healthcare13172108

**Published:** 2025-08-25

**Authors:** Vivian Lüdorf, Sven Meister, Anne Mainz, Jan P. Ehlers, Julia Nitsche, Theresa Sophie Busse

**Affiliations:** 1 Didactics and Educational Research in Health Care, Department of Human Medicine, Faculty of Health, Witten/Herdecke University, 58455 Witten, Germany; 2 Health Informatics, Department of Human Medicine, Faculty of Health, Witten/Herdecke University, 58448 Witten, Germany; 3 Department of Healthcare, Fraunhofer Institute for Software and Systems Engineering ISST, 44147 Dortmund, Germany; 4 Digital Health, Department of Human Medicine, Faculty of Health, Witten/Herdecke University, 58455 Witten, Germany

**Keywords:** healthcare data management, data literacy, outpatient healthcare, digital transformation, ELSI concept, ELSA concept, ethics, social responsibility, professional competencies, informed consent, ELSI, ELSA

## Abstract

(1) **Background:** Data literacy is becoming increasingly important for healthcare professionals in both outpatient care and research. Since healthcare data and the possibilities for its use and misuse are increasing in these areas, healthcare professionals need diverse knowledge regarding the collection, use and evaluation of data. A core component of this is an understanding of the ethical, legal, and social implications (ELSI) of working with health data. (2) **Methods:** Within the DIM.RUHR project (Data Competence Center for Interprofessional use of Health Data in the Ruhr Metropolis), the challenge of training in data literacy for different healthcare professionals is addressed. Based on a learning objectives matrix for interprofessional data literacy education, an ELSI concept was developed through collaboration with interprofessional project partners. The study was conducted between December 2024 and April 2025. (3) **Results:** The foundational structure of the ELSI concept was based on the learning objectives matrix and an unstructured literacy search for ELSI concepts in similar contexts. Using an iterative design-based research approach, a group of experts from different fields (didactics, applied ethics, health sciences, law, sociology, informatics, and psychology) developed an ELSI concept for healthcare professionals. The following categories were identified as crucial: 1. philosophy of science: a basic understanding of science and the hurdles and opportunities; 2. ethics: an overview of the biomedical principles and a technological assessment; 3. law: an overview of the reservation of permission and self-determination; 4. social aspects: an overview of health inequalities and different forms of power relations and imbalances. (4) **Conclusions:** The ELSI concept can be used in the orientation of healthcare professionals in outpatient care and research—regardless of their profession—to develop data competencies, with the aim of providing a holistic view of the challenges and potential in the collection, use, and evaluation of healthcare data. The DIM.RUHR project’s approach is to develop open educational resources that build on the ELSI concept to teach specific skills at different competence levels.

## 1. Introduction

In the era of digital transformation, healthcare data are increasing in volume, complexity, and significance [1]. Professionals in different fields of healthcare are therefore increasingly confronted with data-driven decisions on a daily basis. For healthcare professionals, digital technologies, data-driven processes, decision-making, and large volumes of healthcare-related data require well-grounded data competencies to effectively cope with the constantly evolving challenges of digital workflows [2]. In outpatient settings, large amounts of healthcare data are routinely generated, for instance, through electronic health records, diagnostic procedures, medication plans, and digital health applications [3,4]. In inpatient settings, data from prescription and pharmacy documentation is used to support antimicrobial stewardship [5], while data from electronic health records can improve decision-making [6]. To make sound medical decisions, professionals must be capable of managing digital systems competently and storing, structuring, and analyzing data appropriately [7]. The effective handling and use of health data also plays a vital role in research contexts to find and address challenges regarding data collection and management [8,9]. In order to continuously enhance data quality, professionals working at the intersection of clinical care and research need to understand how data is accurately collected, analyzed, and interpreted within the concept of evidence-based medicine [10].

Data literacy can be understood as a meta-competence that unites a variety of areas and is essential regarding the increased use of data in healthcare. The working definition of data literacy used in this study is based on a comprehensive literature review conducted for the development of a learning objectives matrix for interprofessional data literacy education in outpatient healthcare and research [11]. This work was carried out as part of the DIM.RUHR (Data Competence Center for the Interprofessional Use of Health Data in the Ruhr Metropolis) project, which also included the development of the ELSI concept presented here. The learning objectives matrix draws primarily on the definitions proposed by Ridsdale et al. [12], Grillenberger and Romeike [13], and Schüller [14]. According to Ridsdale et al. [12], data literacy is defined as the ability to collect, manage, evaluate, and apply data. Furthermore, data literacy encompasses the knowledge, skills, and values necessary for the effective planning, execution, and improvement of all steps involved in deriving value or making decisions from data under ethical, legal, and social considerations [12,13,14].

The necessity of adequately addressing these continuously growing challenges requires not only a deep understanding and well-founded application of health data but also its ongoing critical evaluation by healthcare professionals [15]. In this context, attention is often predominantly focused on the technical and analytical skills of those involved in the collection, interpretation, assessment, and application of data [16]. However, critical and reflective data literacy plays a central role, referring to the ability to continuously reflect on data practices and evaluate them within their broader societal context, beyond mere technical execution [17]. In education and training programs aimed at promoting responsible health data practices, the systematic integration of a holistic understanding of data use as a dimension of professional responsibility is frequently lacking [18]. A lack of holistic data literacy can lead to violations of data protection regulations and ethical and social standards, and may result in biased or distorted data interpretation [19,20]. A purely technical perspective therefore fails to account for the high sensitivity of health data, which is inherently linked to individual rights, ethical principles, and social values [21]. In light of these considerations, attention to ethical, legal, and social implications (ELSI) represents a necessary extension of data literacy in the healthcare sector [22].

### 1.1. Do Data Competencies Require ELSI Skills?

Data literacy in healthcare requires that health professionals possess ELSI-related competencies in order to ensure the responsible handling of data and to critically reflect on and evaluate their own practices within data-driven processes [23]. Engaging with data in healthcare inherently constitutes a direct or indirect intervention in individuals’ lives and is therefore accompanied by an ongoing responsibility [24]. Data often reflect and reinforce existing social inequalities and are deeply intertwined with ethical, legal, and social considerations [25]. This is particularly crucial in the healthcare sector, where sensitive information, critical health-related decisions, and patient trust are at stake. An ELSI concept can help to address these challenges.

The ELSI concept in the present study refers to a structured approach designed to ensure that data-driven processes in healthcare are not only technically effective but also grounded in ethically, legally, and socially acceptable principles. From an ethical perspective, the ELSI concept considers what constitutes responsible data use, how patient autonomy over personal data can be safeguarded, and how concerns such as bias, fairness, and transparency in data practices should be addressed [26]. From a legal perspective, it involves determining which laws and data protection regulations apply and how to strike an appropriate balance between protecting personal data and enabling innovation and knowledge generation, particularly in health research [26]. The social dimension focuses on the unequal treatment of population groups in the context of health data, which often results from structural discrimination and systemic inequality [26].

The overarching goal of ELSI-informed research practices for data literacy in healthcare is to facilitate the early identification of potential risks and opportunities, and to strengthen public trust, equity, and inclusion [27]. Strategies in embracing data-driven healthcare provide a foundation for ethically responsible action among stakeholders in care, research, and policy [28]. This applies especially to the continuous safeguarding and assurance of data protection, individual patient rights, and awareness of the susceptibility to errors and biases in data [29,30]. A lack of understanding of ELSI in the handling of health data is therefore problematic for various reasons and can manifest in multiple contexts. The following examples illustrate current and prominent possible scenarios in which profound ELSI competencies are essential when dealing with health data:

- Medical research.
- Artificial Intelligence (AI)-assisted medical diagnostics and triage systems.
- Electronic Health Records, telemedicine and online consultations.
- Genomic databases and personalized medicine.
- Care robots and sensor-based monitoring systems in nursing homes.
- Digital health applications for self-monitoring.
- Wearables, such as smartwatches and fitness trackers.

### 1.2. Teaching of Healthcare Professionals Regarding ELSI

In healthcare, robust ELSI competencies are essential in various professional contexts. While the specific focus of these competencies may differ depending on the professional role, the core dimensions of ELSI are relevant across all healthcare professions. This is particularly evident in digital or AI-supported data processes, where a comprehensive understanding of informed patient consent is required from all healthcare professionals [31]. Moreover, healthcare professionals, especially those involved in patient care and research, are often in close contact with diverse populations, including vulnerable groups [32]. As such, they require a deep understanding of social and ethical considerations in their daily practice [33]. What additionally unites all healthcare professions is their shared responsibility to ensure the proper handling of legal data protection issues and to engage in ethically sound communication with patients [34]. ELSI competencies in the use of health data therefore represent a highly relevant component of professional competence in healthcare.

Despite the critical importance of promoting an ethical, legal, and socially responsible approach to health data, ELSI has not yet been fully integrated into education and training programs in the healthcare sector [35]. Curricula and training regulations tend to emphasize the technical aspects of data handling, while placing considerably less focus on reflective and critical dimensions [17]. Although interest in the topic is growing, there remains a lack of standardized teaching models for incorporating ELSI into the development of data literacy in healthcare. A systematic integration of ELSI into educational and training structures is therefore not only desirable but also urgently needed in order to foster data literacy based on ethical, legal, and social principles.

### 1.3. Objectives/Conclusions

Data literacy must thus be recognized as an integrative competence, encompassing the ability to work with data in alignment with technical as well as ethical principles, legal requirements, and societal expectations [36]. Such a holistic conception of data literacy enables healthcare professionals not only to utilize technology effectively, but also to actively contribute to the development of a responsible, human-centered, and sustainable healthcare system [26,31].

As part of the DIM.RUHR (Data Competence Center for Interprofessional Use of Health Data in the Ruhr Metropolis) project, launched in 2023 (grant number: 16DKZ2008A-F), funded by the German Federal Ministry of Research, Technology and Space (Bundesministerium für Forschung, Technologie und Raumfahrt; BMFTR) and the European Union (NextGenerationEU), a concept was developed that focuses on the ELSI involved in promoting data literacy among healthcare professionals. It builds on a learning objectives matrix developed within the project by Lüdorf et al. [11], which itself is based on the learning objectives matrix for research data management by Petersen et al. [37]. The aim is to promote interprofessional data literacy in outpatient care and health research by providing learning modules, which will be made available as Open Educational Resources (OER).

The ELSI concept serves as the foundation for the ethical, legal, and social orientation of these modules and is intended to enable healthcare professionals to engage with health data responsibly and reflectively. This study presents the development of the ELSI concept as a basis for teaching and learning data literacy in healthcare. It can also support ethical decision-making when collecting, using, and evaluating data in both clinical and research settings. The concept helps ensure that both the challenges and potentials of health data use are recognized and addressed by relevant stakeholders. Moreover, it can be applied in various contexts where ethical, legal, and social considerations are essential for sound judgment and responsible practice. In contrast to existing models that often address ethical, legal, or social aspects in isolation, the ELSI concept offers an integrated and practice-oriented approach that brings these dimensions together within a coherent educational framework. Its design explicitly supports curricular implementation across diverse healthcare settings and professions, while remaining adaptable to different institutional and regulatory contexts. This integrative and application-focused orientation constitutes a central innovation of the concept.

## 2. Materials and Methods

### 2.1. Development of the ELSI Concept

The ELSI concept was developed between December 2024 and May 2025 as part of the DIM.RUHR project, where it serves as an overarching foundation for the creation of OER aimed at promoting data literacy in outpatient healthcare and research. Within the project, the concept is intended to provide healthcare professionals with essential, low-threshold knowledge regarding the ethical, legal, and social implications of health data use, enabling them to recognize challenges and reflect on responsible data practices. Although developed in the context of DIM.RUHR, the ELSI concept also offers broader applicability. It may serve as a foundational framework for other healthcare professions, disciplines, and institutions engaged in data literacy and health data education, particularly where there is a need to introduce ELSI-related topics at a basic competency level.

The development of the ELSI concept began with the formation of a dedicated working group in November 2024. A multidisciplinary team consisting of various experts in applied ethics, social sciences, laws, didactics, health informatics, and healthcare practice collaboratively designed the concept, starting with a first meeting in December 2024. Its development was informed by expert judgment and discipline-specific knowledge, enabling an inductive conceptual foundation. The process followed an iterative model that incorporated multiple rounds of review and feedback. Revisions were undertaken based on internal discussions and bilateral consultations. This ensured reflexive integration of the various multidisciplinary perspectives and didactic alignment while indicating a consensus-oriented approach. Disciplinary contributions were taken into account as well as the perspectives of healthcare professionals in research and care with different competency levels.

This study draws selectively on elements of the design-based research approach [38], without fully adhering to all of its methodological steps. This approach is typically situated in real-world educational contexts and focuses on the design and iterative testing of meaningful interventions [38]. It is characterized by the use of mixed methods, collaborative engagement between researchers and practitioners, and multiple cycles of refinement [38]. A central feature of the design-based research approach is the derivation of design principles through grounded theorizing. In the present study, this aspect was addressed by a multidisciplinary project group composed of both former and current practitioners from various healthcare fields, as well as university educators in health professions. This collaborative foundation contributed to the theoretically grounded development of the learning objectives matrix. While the study does not constitute a full implementation of the approach, elements such as the practical relevance of the intervention and its alignment with action research traditions were considered. Additionally, the use of personas, which were previously established in other phases of the project, proved useful in addressing the needs and practices of diverse target groups.

**Figure 1:**
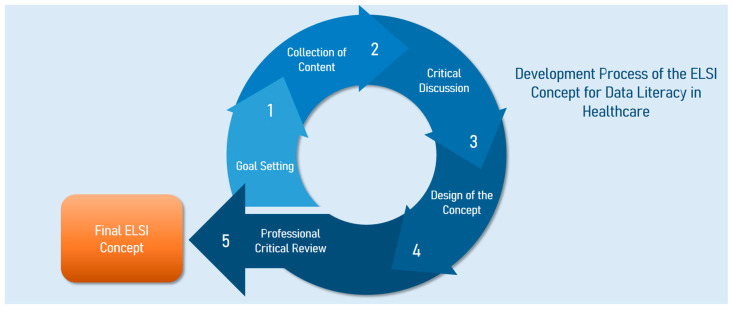
Illustration of the individual stages in the ELSI concept development.

The individual steps involved in the development of the ELSI concept are outlined below and are subsequently summarized in Figure 1 at the end of the chapter, which provides an overview of the entire process.

#### 2.1.1. Goal Setting

In December 2024, a first meeting was held to discuss the content as well as the structure of the ELSI concept. All participants brought their experience from their field as well as knowledge from their unstructured literature searches within their respective domains to gather background information and identify existing ELSI concepts. Previously, participants were divided based on their expertise into those with a more global focus, those with a focus on law, those with a focus on social aspects, and those with a focus on ethical aspects. This was developed as an inductive qualitative procedure.

#### 2.1.2. Initial Collection of Content in the Structure of the Learning Objectives Matrix

The contents of the ELSI concept, including the associated knowledge, skills, and value-based attitudes, were developed for each thematic area based on the learning objectives matrix by Lüdorf et al. [11] in January 2025. This matrix outlines key competence areas necessary for data-literate practice in healthcare professions. It distinguishes eight subject areas that collectively represent the essential dimensions of data literacy in this context: (1) fundamentals and general concepts; (2) ethical, legal, and social aspects; (3) building a data culture; (4) data acquisition; (5) data management; (6) data analysis; (7) data interpretation; and (8) deriving measures. Within these areas, health professionals’ data literacy can be assessed at four levels of competence: basic, advanced, highly specialized, and very highly specialized [11]. These competencies are essential not only for delivering high-quality, patient-centered care but also for ensuring the efficient operation of data-related processes [39].

The structure was used to offer the best possible representation of different skill levels and knowledge. A tabular structure was therefore chosen, distinguishing between different competencies and competency levels. In this phase, an initial draft of the foundational design was created by one participant of the group based on the previous exchange, incorporating first only ethical considerations. This approach was chosen in order to establish a common basis and to examine possible ways of shaping this diverse topic. Feedback was obtained on the developed draft of a tabular structure of ethical aspects by the internal group to ensure alignment with the overall concept of data literacy, and the draft was subsequently revised by the initial creator to reflect this input. Further review focused on ensuring consistency with the learning objectives and improving the structural clarity of the concept. In the next step, the revised version was used as the basis for developing specific drafts addressing the social and legal dimensions, respectively. Here, too, a tabular structure was chosen.

#### 2.1.3. Critical Discussion Regarding the Target Group, Persona and Competence Level

In a follow-up meeting held in January 2025, the draft versions of the tables on each topic were discussed by the full group. Particular attention was given to the levels of competency, as well as the scope and objectives of the concept. A separate focused discussion was held to explore the interrelation between social and ethical aspects, along with considerations from the philosophy of science. Building on the outcomes of the group discussion, the main structure of the ELSI concept was further refined. To ensure alignment across all competency levels, adjustments were made to both the depth and structural organization of the concept. It was decided that the ELSI concept should initially map the lowest level of competence relevant to all health professions. The ELSI concept can thus best serve as a knowledge base, while the OER to be developed for the named subject areas (Section 2.1.2) should impart skills and knowledge that build on this and thus cover the higher competence levels on the basis of the learning objectives matrix.

In this context, the relationship between the ELSI dimensions and the overarching data literacy competencies was further clarified. Each of the ELSI dimensions intersects with key areas of data literacy in distinct ways. For example, ethical considerations are particularly relevant in data acquisition (e.g., informed consent), legal dimensions play a central role in data management (e.g., data protection regulations), and social implications become prominent in data interpretation (e.g., addressing bias or fostering public trust). While these categories are not rigidly tied to specific educational levels, their complexity and required depth of engagement increase progressively from basic to advanced levels of professional development. As the ELSI concept is explicitly designed to address the basic level of data literacy competence applicable across all health professions, the focus lies on providing a foundational orientation rather than a full mapping across all levels.

The revised approach was subsequently discussed in smaller working meetings to gather additional feedback and refine the integration of perspectives from different thematic areas.

#### 2.1.4. Design of the ELSI Concept

Building on the tabular elaborations of the three thematic focal points, structured according to the logic of the learning objectives matrix, the ELSI concept was further iteratively developed from February to March 2025 to serve as an introduction to these dimensions within the context of data-related competencies. The document was subsequently reorganized to improve coherence, with the section on social aspects further elaborated. In order to achieve this, the participants chose to depart from the original structure and opted for a continuous-text format. They concluded that, particularly in light of the omission of the higher competence levels, this approach offered the clearest and most effective means of presentation. The ethical component was revised and partially rewritten to align with the lowest level of competency and to ensure consistency with the social dimension. The legal aspects were added and integrated accordingly. The overall structure and content were reviewed for alignment with the learning objectives matrix and didactic framework. An introductory text was also drafted to emphasize the importance of scientific theory and highlight the objectives of the ELSI concept. A final collaborative meeting was held to consolidate the contributions and finalize the document. The completed version was then reviewed for quality and coherence by additional members of the team.

#### 2.1.5. Professional Critical Review of the ELSI Concept

The whole consortium was then asked for feedback on the concept in April 2025. By involving the whole consortium, the transfer between theory and practice was carried out in this step. The inclusion of all participants serves above all to test the resulting ELSI concept for understanding and practicality in use.

## 3. Results

The full English translation of the ELSI concept is provided as Supplementary Material S1; it was developed in German.

### 3.1. Target Audience

The content of the ELSI concept primarily targets professional groups in outpatient healthcare, who the project will address. These groups were previously represented during an initial project phase through the development of five distinct personas. These personas were designed to comprehensively reflect the needs and challenges faced daily by the target groups within the outpatient healthcare professions and to construct realistic user profiles. As defined by Lüdorf et al. [11], the personas representing the target groups include general practitioner, medical student, student of medical professions (e.g., healthcare, nursing science, midwifery science), medical assistant, and young researcher. Each of these personas is associated with specific ethical, legal, and social considerations in their engagement with health data. For example, general practitioners must navigate issues such as informed consent, access rights, and professional responsibility in data handling. Medical assistants are more likely to face questions of documentation, confidentiality, and data exchange within teams. Medical and nursing students require introductory guidance on ELSI principles relevant to digital practice environments. Young researchers, on the other hand, are confronted with research ethics, anonymization, and secondary use of data.

The ELSI concept is intended to serve as a cross-cutting orientation providing guidelines for how data literacy should be conveyed to the target groups, defined through these personas, via OER. Consequently, the OER will be developed based on the principles and structure of the present ELSI concept. Beyond addressing the healthcare professions themselves, the ELSI concept also serves as a guideline for the project team, which is expected to act in accordance with ethical, legal, and social principles.

The ELSI concept is primarily aimed at the aforementioned healthcare professions, who typically possess only limited prior knowledge and fundamental competencies in handling data. Accordingly, the content is intentionally maintained at a basic level, as the goal is not to impart expert knowledge but rather to communicate essential ethical, legal, and social issues that may arise in the handling of health-related data.

Although the concept is tailored to the specific professional groups involved in the project, it can also be applied beyond this scope. It holds relevance for a wide range of healthcare professions as well as other disciplines and institutions engaged in data literacy and health data practices.

### 3.2. Structure

The present ELSI concept is structured into four chapters: Introduction, Ethics in the Use of Health Data, Social Aspects, and Legal Foundations. While each chapter, apart from the introduction, can be read independently, they are nonetheless conceptually interlinked and built upon one another. At the end of each chapter, clear and actionable recommendations are provided in the form of a concluding summary. Furthermore, a deliberate decision was made to deviate from the conventional sequence of ethical, legal, and social sections. In this concept, the legal chapter is intentionally placed last, as it conceptually builds upon the preceding discussion of ethical and social aspects, particularly because the protection of legal rights is essential to upholding both ethical standards and social equity.

### 3.3. Content

#### 3.3.1. Relevant Ethical Aspects

The use of health data in outpatient care and research raises a wide range of ethical questions. These issues are not only highly sensitive but also require ongoing evaluation in light of societal and technological developments. The increasing use of artificial intelligence and automated processes introduces numerous ethical challenges and places considerable responsibility on all stakeholders, including healthcare professionals, researchers, and policymakers. A responsible approach to managing health data requires adherence to the fundamental principles of medical ethics, as well as the use of structured methods for ethical reflection. The approaches presented in this section of the ELSI concept offer methodological guidance for promoting ethically sound data practices. To support this, the principlism framework developed by Beauchamp and Childress [40] is applied. This model is grounded in a shared moral understanding and is based on four widely recognized bioethical principles: respect for autonomy, non-maleficence, beneficence, and justice. These principles have been adapted in the present ELSI concept to guide the handling of health data. According to Beauchamp and Childress [40], the principle of respect for autonomy involves acknowledging individuals’ autonomous decisions and supporting their capacity for autonomous decision-making [40]. In the context of health data, this entails recognizing individuals’ right to data sovereignty and their right to make informed and self-determined decisions about the use of their personal health data. Informed consent should be obtained through transparent communication about data use and must be voluntary and continuously updated in accordance with evolving data governance frameworks. The principle of non-maleficence implies that harm to individuals or groups must be prevented [40].

This can be transferred to the use of health data. Privacy risks and the potential misuse of sensitive health data must be consistently considered, and robust technical and organizational safeguards are essential to ensure data protection. The principle of beneficence emphasizes the positive obligation to support and assist others [40]. Health data should be actively used to enhance patient and societal well-being—for example, in the areas of prevention, diagnosis, and treatment. Striking a balance between data protection and innovation is essential to simultaneously uphold individual rights and harness the societal benefits of health data, such as enabling more efficient research and healthcare delivery. The principle of justice refers to the fair distribution of resources, rights, and responsibilities [40]. In the context of health data, this requires removing structural, technical, and linguistic barriers to ensure equitable and non-discriminatory access to healthcare and research. It is crucial to ensure that all population groups are adequately represented in datasets to prevent inequality and bias.

In addition to these ethical principles, continuous evaluation and technology assessment are vital. The latter involves analyzing the social, ethical, and ecological impacts of technologies and assessing their potential risks and benefits [41]. This process informs the participatory and interdisciplinary development of ethical guidelines, involving all relevant stakeholders from healthcare, research, and policy.

#### 3.3.2. Relevant Social Aspects

The relevant social aspects addressed in the present ELSI concept are derived from societal norms and values, which in turn shape actions and interactions. A key issue in this context is health inequality, which arises from social and structural disparities such as gender, socioeconomic status, and ethnic background. According to Elkeles and Mielck [42], social disparities influence health inequalities through three main factors: access to healthcare services, exposure to health-related burdens, and coping capacities. The particular importance of intersectionality, describing the simultaneous overlap of various forms of discrimination, must also be emphasized [43,44].

Translating these concerns into the responsible use of health data, the ELSI concept emphasizes that health data must be collected, analyzed, and used in a manner that ensures no individual is disadvantaged based on gender, social or ethnic background, disability, or other characteristics. In research contexts, this necessitates the adequate inclusion and high representativeness of all population groups, particularly those considered vulnerable. Health data should be leveraged to identify inequalities between groups and inform more equitable healthcare provision. This also includes actively involving patients in decisions concerning their health data and raising awareness of their rights. Given that algorithmic bias can significantly distort existing health inequalities and produce discriminatory outcomes, ongoing evaluation and adjustment of such systems are essential to ensure fair and non-discriminatory decision-making.

An unequal power dynamic between researchers and research participants can also play a critical role in reinforcing social inequities in healthcare [45]. This includes the risk that participants may not be adequately informed about the potential risks and consequences of a study or may feel pressured to participate. To address this, the ELSI concept calls for transparent and comprehensible information to be provided to research participants. Comprehensive education, active participant involvement, and participatory research approaches are essential to fostering a fair and respectful research environment. Such power imbalances are also reflected in severe violations of data privacy and the misuse of personal data, which can cause significant social harm to participants, including discrimination, stigmatization, and exclusion. Therefore, it is vital to maintain a careful balance between research benefits, individual patient rights, and innovation. Upholding ethical standards requires transparent decision-making processes, independent oversight bodies, and the stronger involvement of patients in research processes. Identifying and critically reflecting on potential biases is crucial to avoid erroneous conclusions and prevent the reproduction of social inequalities in research outcomes.

#### 3.3.3. Relevant Legal Aspects

As previously outlined, the chapter on legal foundations in the present ELSI concept is intended to build upon and integrate the preceding ethical and social considerations. It is grounded in German jurisprudence and reflects the national legal context within which health data are collected and processed, and is therefore shaped by constitutional principles, data protection laws, and relevant case law in Germany. It is crucial to bear in mind that the law, particularly data law, is subject to constant change in Germany due to new technical developments. This was a major challenge in the development of the ELSI concept. Given the multitude of legal regulations governing the use of health data, the specific context is particularly decisive. For example, the applicable data protection law may vary depending on the responsible institution, such as religiously affiliated hospitals or social insurance carriers that provide routine data. Nevertheless, overarching all specific regulations is constitutional law, from which consistent principles for the formulation of individual statutes can be derived. The legal starting point is primarily situated within the tension between the continuous safeguarding of data protection and the pursuit of scientific knowledge. According to legal doctrine, this is fundamentally based on the general right to personality, which encompasses the fundamental right to informational self-determination. This means that individuals, i.e., patients, have the right to decide how their personal data is handled. The governing principle in this context is prohibition with the reservation of permission, which stipulates that each instance of data use must have an explicit legal basis allowing for that specific use.

In the context of health data, the principle of purpose must also be observed. This principle requires that the law governing data collection clearly and precisely defines the purposes for which the data may be collected and further processed. If the legislature wishes to allow data use beyond the original purpose, a separate legal basis must be established. Fundamentally, any infringement on the right to informational self-determination must always be legally justified and proportionate. Although the enabling provisions typically include requirements for the informed consent of the data subjects, it is not excluded that data may also be used without the patient’s consent. This depends on compliance with the principle of proportionality, which requires that the data processing serves a legitimate aim and is appropriately carried out. In such cases, the restriction of the right to informational self-determination must be weighed against the purpose of the data processing. Particular importance is attached to the implementation of data anonymization and/or pseudonymization. Blanket data collection without a specific purpose (data retention) is not permitted. An interference with the right to informational self-determination is more likely to be justified if it results in a socially valuable gain in knowledge. Therefore, it is essential that the research project adheres to the principles of good scientific practice and incorporates relevant ethical and social considerations.

## 4. Discussion

To meet the growing demands of digital transformation and to address the associated challenges effectively, healthcare professionals require robust data literacy [1]. A well-grounded understanding of data practices contributes significantly to the efficient functioning of healthcare systems and supports both patient-centered care and health research [39,46]. Data literacy involves not only technical and analytical skills but also critical–reflective competencies that contextualize data use within broader social, legal, and ethical frameworks [17]. Specifically, the handling of sensitive health data demands informed, responsible action from all stakeholders, grounded in a comprehensive awareness of the ethical, legal, and social implications inherent in data-driven processes [35].

Building on this rationale, the present study seeks to develop a conceptual ELSI concept for data literacy that addresses the specific needs of healthcare professionals. In a five-step process, a small group first developed a concept based on a learning objective matrix for data literacy in healthcare professions by Lüdorf et al. [11]. This concept is designed within the context of the DIM.RUHR project. It aims to provide a theoretical foundation for the design of OER that promotes interprofessional data literacy grounded in ethical, legal, and social principles. The ELSI concept was then critically reviewed and further developed by experts from professional practice, research, and teaching. The development process was particularly challenging in terms of determining the required skill levels in such a concept, linking the various aspects, establishing the relevance of scientific theory as a possible basis, and addressing the changeability of legislation. The process was guided by the framework of design-based research [38].

The relevance of data literacy in healthcare from an ELSI perspective becomes particularly evident in contexts where informed decisions must be made when there are tensions between technical feasibility, ethical responsibility, legal requirements, and societal acceptance. This is exemplified by the handling of health data during the COVID-19 pandemic, in which large volumes of sensitive patient data were collected for purposes such as contact tracing, vaccination statistics, and research [47]. In this context, data literacy as understood within the ELSI concept was especially important among all stakeholders to identify risks to privacy and data protection, enable informed consent, and foster public trust by designing socially acceptable solutions [47].

Gray et al. [48] developed a definition of data infrastructure literacy in 2018 and argued even then that data literacy initiatives need to promote awareness not only of data science, but also of data politics and data sociology, as well as broader public engagement with digital data infrastructures. In addition to the sociological skills explicitly required here, the concept presented enables required actions to be taken in political contexts and public engagement due to its ethical and legal foundations.

Another illustrative case is the use of AI-based diagnostic systems, which raise ELSI-related concerns regarding the transparency of algorithmic decisions, liability in cases of misdiagnosis, and potential discrimination against specific population groups [31]. Here, too, healthcare professionals and institutions can only adopt and apply such technologies responsibly and effectively if they possess adequate data literacy informed by ELSI considerations [31].

Despite its growing importance, data literacy remains insufficiently embedded in many healthcare education curricula [18]. Where data-related content is included, it often concentrates on technical competencies such as the use of electronic health records or basic data management [21]. However, critical skills for working with health data are frequently lacking [49], such as the ability to assess data quality, interpret algorithmically generated outputs, question the provenance of data, and address patients’ concerns about privacy and surveillance.

Moreover, existing approaches rarely integrate data skills into real-world decision-making contexts that involve uncertainty, bias, or conflicting values. This results in a disconnect between formal training and the complex, value-laden situations that healthcare professionals encounter in practice. For example, while practitioners may be taught to obtain informed consent in a procedural sense, they are often not encouraged to critically reflect on whether consent is truly informed, especially in the context of extensive data-sharing or AI-supported diagnostics [50]. Similarly, there is limited focus on identifying and mitigating discriminatory risks embedded in datasets or algorithmic decision systems, particularly those affecting marginalized or underrepresented populations [23,26].

As an integral part of the lifecycle of data processes, data literacy should therefore be understood as an integrative competency that situates technical proficiency within a solid ELSI concept [51]. This holistic understanding enables healthcare professionals in both clinical and research settings to engage with data in a responsible, informed, and ethically sound manner [28]. To achieve this, it is essential that such integrative content is incorporated into healthcare education and training from the outset.

### 4.1. Implementation Strategies

The concept is designed to support healthcare professionals in engaging with health data in a responsible and reflective manner. It aims to foster ethically sound decision-making throughout data-related processes in both clinical practice and research. By addressing the challenges and opportunities of health data use, the concept encourages the involvement of relevant stakeholders and emphasizes the importance of contextual sensitivity.

To comprehensively implement ELSI considerations in the education and training of healthcare professionals, various didactic strategies could be integrated into learning processes. Evidence-based simulation scenarios in healthcare settings have proven to be effective educational tools [52]. Although interdisciplinary case studies specifically addressing ELSI in the context of data literacy in healthcare are not yet well established, they could hold promise as a valuable method for developing such competencies by engaging learners in realistic and complex practice-based situations. Likewise, working with AI-driven diagnostic tools [31] or engaging in structured reflection [53], where learners relate data practices to patient rights, discrimination risks, and issues of social justice, could help foster awareness of ELSI dimensions in the use of health data as well. Due to the large number of people who did not have access to this topic during their education, continuing education formats can be particularly useful. Egbert et al. [29] report on a summer school in which they were able to strengthen the critical thinking, problem-solving skills, and interprofessional and intercultural competencies of healthcare professionals through a digital preparation phase and a five-day face-to-face event focusing on group work, problem-solving strategies, and data literacy.

To ensure curricular integration, ELSI-related content may be embedded in existing modules such as ethics, law, or digital health, and aligned with national learning objectives and competency frameworks. It can be assumed that integration into existing modules and content is possible, thus enabling a multi-perspective expansion. Depending on the educational level, this can include foundational courses for undergraduates as well as elective or interprofessional modules for advanced learners. Vertical integration across the curriculum can support the progressive development of competencies, from basic knowledge to applied ethical reasoning.

Although developed within the DIM.RUHR project, the concept’s applicability extends beyond this specific context. It may also serve as a resource for educators and institutions in other healthcare settings or disciplines seeking to develop data literacy curricula aligned with ELSI principles. To enhance the adaptability of the ELSI framework across different legal and institutional contexts, the concept follows a modular and principle-based approach. While the legal dimension is grounded in the German regulatory environment, the underlying principles, such as purpose limitations, proportionality, and data minimization, are widely recognized in international data protection frameworks and can therefore be considered transferable. Rather than prescribing specific legal norms, the framework highlights normative legal concepts that can be contextualized according to national legislation, such as the EU General Data Protection Regulation (GDPR) or the U.S. Health Insurance Portability and Accountability Act (HIPAA).

In practical implementation, this allows for the substitution or expansion of jurisdiction-specific content in educational modules and invites collaboration with local legal experts or the integration of comparative case studies. By maintaining stable ethical foundations while allowing for legal adaptation, the ELSI framework offers a flexible and future-oriented structure that can support localized implementation without losing coherence or conceptual integrity.

### 4.2. Limitations

Although the ELSI concept aims to adequately reflect potential ethical, legal, and social challenges, several limitations of the present study must be acknowledged. One key limitation is the absence of empirical testing in real-world healthcare environments. While this concept serves as the conceptual foundation for the development of interprofessional learning modules in the form of OER, these materials are still under development and have not yet been subject to formal evaluation.

Moreover, the concept constitutes a theoretical construct and does not claim to provide an exhaustive account of all ethical, legal, and social dimensions of data use. ELSI-related processes are inherently complex and highly context-dependent. Legal aspects, as mentioned above, are particularly subject to continuous change and often require precise definitions to be meaningfully addressed. This also applies to ethical and social issues: although overarching scenarios can be identified, the challenges arising in the context of health data use must be continually reassessed and critically examined. The ethical foundation of the framework is based on the principle-oriented approach by Beauchamp and Childress, which offers a robust and well-established basis for ethical reflection in biomedical contexts. However, this foundation represents only one possible ethical perspective. Alternative approaches, such as virtue ethics, care ethics, or deontological frameworks, may provide valuable additional viewpoints, particularly for specific professional groups or educational settings. Future adaptations or extensions of the framework may, however, benefit from incorporating such complementary ethical theories to broaden its applicability and complement diverse perspectives and approaches.

In addition, the implementation of the ELSI concept involves specific challenges from an information science perspective. Healthcare professionals vary considerably in their baseline levels of data literacy, which may impact the uptake and perceived relevance of ELSI content. Translating abstract ethical and legal principles into accessible, role-specific OER formats requires careful pedagogical adaptation. Moreover, both technological advances and evolving ethical–legal standards necessitate continuous revision of educational materials to ensure long-term curricular relevance. Accordingly, the ELSI concept provides a foundation for critical reflection and aims to support the responsible development of educational materials in the context of healthcare data literacy.

### 4.3. Outlook and Implications

As the interprofessional learning modules for data literacy are currently being developed within the DIM.RUHR project, the ELSI concept is actively being used in this process. However, a comprehensive evaluation of whether the resulting OER fully aligns with ELSI principles is still pending. To ensure the practical relevance and effectiveness of the proposed ELSI framework, an empirical evaluation phase is planned. Several OERs developed within the project will be implemented and tested in real-world healthcare education settings. Specifically, these pilot implementations will take place in university courses as well as in outpatient care environments, such as general medical practices. Their evaluation will primarily rely on pre- and post-intervention surveys to measure changes in knowledge, attitudes, and ethical reasoning, as well as semi-structured interviews with learners and educators to gain deeper insight into the usability, relevance, and perceived impact of the OER materials. These findings will provide essential empirical feedback for the further development and refinement of both the ELSI framework and the associated educational resources for data literacy development.

Additional modules focusing explicitly on ethics, social aspects, and law are also being developed as extensions of specific competency levels. Despite not being exhaustive, the concept may serve as a valuable orientation tool beyond the project’s scope. It can assist educators and content developers in creating teaching and learning materials that integrate ethical, legal, and social considerations into data literacy education in healthcare contexts. Furthermore, the concept can help highlight critical challenges within healthcare data practices and suggest possible courses of action. In doing so, it supports ongoing reflection and engagement with the broader implications of health data use.

## 5. Conclusions

The development of a concept for integrating data literacy in healthcare through an ethical, legal and social lens represents a significant advancement in conceptualizing data competencies beyond purely technical domains. Challenges in developing ELSI concepts for similar use can include determining the required skill levels, linking the aspects regarding ethics, society, and law, deciding on the relevance of scientific theory, and addressing the changeability of legislation as well as all other aspects. By embedding critical and reflective dimensions into data literacy, this concept aims to empower healthcare professionals to navigate the complex challenges arising from the use of health data in a rapidly digitizing care landscape. This approach promotes the continuous critical evaluation of data-related processes and fosters the responsible, secure, and inclusive handling of health data among all actors involved in care and research. It contributes to a foundation for ethically informed and patient-centered healthcare that aligns with core legal and societal values.

To support this transformation, targeted action is required at multiple levels. Educational institutions should integrate interdisciplinary ELSI modules into the healthcare curricula, policymakers are encouraged to establish supportive regulatory and funding frameworks, and professional bodies should define ELSI-oriented competencies as essential components of digital literacy standards.

The future integration of such an ELSI concept into education and training for healthcare professionals will be essential to improving the quality and integrity of healthcare and research. It will also contribute to safeguarding patient rights and addressing structural inequalities. Further research and broader awareness of ELSI considerations are therefore indispensable for shaping a digitally enabled healthcare system that is both responsible and resilient.

## Data Availability

No new data were created or analyzed in this study. Data sharing is not applicable to this article.

## Supplementary Materials

The following supporting information can be downloaded at: https://www.mdpi.com/article/10.3390/healthcare13172108/s1. The full English translation of the ELSI concept is provided as Supplementary Material S1.

## Abbreviations

The following abbreviations are used in this manuscript:

ELSI	Ethical, legal, and social implications
OER	Open Educational Resources
GDPR	EU General Data Protection Regulation
HIPAA	Health Insurance Portability and Accountability Act
