# Supplementary material for: Developing a Concept on Ethical, Legal and Social Implications (ELSI) for Data Literacy in Health Professions: A Learning Objective-Based Approach
Source: Healthcare (Basel). 2025 Aug 25;13(17):2108. doi: 10.3390/healthcare13172108 (PMC12427987; doi:10.3390/healthcare13172108)
Supplement: Supplementary file 1 [file healthcare-13-02108-s001.zip › Appendix_A_ELSI_concept_Healthcare.pdf]

DIM.RUHR

Data Competence Center for Interprofessional Use of Health Data in the Ruhr Metropolis

# Integrated ELSI Concept of the DIM.RUHR Project

**Authors** Theresa Sophie Busse & Vivian Lüdorf

**Contributors** Consortium members of the DIM.RUHR project

**Date** 21.05.2025

**Contact** Consortium management of the DIM.RUHR project
Witten/Herdecke University
dimruhr@uni-wh.de

**License** This work is licensed under the Creative Commons Attribution–ShareAlike 4.0 International License (CC BY-SA 4.0). Organization logos and, where indicated, individual images and visualizations are excluded from this license.

The DIM.RUHR project is funded by the BMFTR under grant number 16DKZ2008A-F and financed by the European Union - NextGenerationEU. The views and opinions expressed are solely those of the consortium and do not necessarily reflect the views of the European Union or the European Commission. Neither the European Union nor the European Commission can be held responsible for them.

With funding from the:

Federal Ministry of Research, Technology and Space

Funded by the European Union
NextGenerationEU

# Purpose of this Document

In the healthcare sector, a substantial volume of data is generated in the context of both research and care. The recently enacted Health Data Use Act (*Gesundheitsdatennutzungsgesetz*, GDNG) promotes the use of health data, while simultaneously placing increased demands on healthcare providers and researchers. Meeting these demands requires data literacy, which is currently neither widely established nor systematically integrated into education and training. Data literacy is a key prerequisite for the quality-assured and data protection–compliant collection, analysis, and interpretation of data, and must be developed in parallel with technical infrastructures such as GAIA-X or the European Health Data Space (EHDS).

The Data Literacy Competence Center DIM.RUHR brings together the expertise of participating research institutions. To make health data usable for health services research, DIM.RUHR develops educational, research, and networking initiatives aimed at strengthening data literacy among researchers and healthcare professionals alike. Practice-oriented learning is supported by existing experimental environments; micro-degree-based programs are provided as open educational resources (OER); and the GesundheitsDatenRepository.RUHR maps the full data life cycle of health data and applies it within data-intensive research projects.

This document is part of a series of project-related publications. Its purpose is to present the ethical, legal, and social implications (ELSI) as a central point of reference for the development and provision of OER within the DIM.RUHR project. The ELSI concept serves as a guiding framework to align the creation of OER with these principles. It is intended as a supportive, non-exhaustive resource that aims to raise awareness of the complex interconnections inherent in the use of health data. Given the thematic complexity, the document outlines possible courses of action but primarily focuses on highlighting key challenges and potentials.

# Content


1. Introduction ........ 1
2. Ethics in the Use of Health Data ........ 3
   - 2.1 Principlism by Beauchamp and Childress ........ 3
   - 2.2 Balancing of Principles ........ 4
   - 2.3 Technology Assessmen ........ 5
   - 2.4 Conclusion ........ 5
3. Social Aspects ........ 6
   - 3.1 Power Imbalances between Researchers and Participants ........ 9
   - 3.2 Scientific Benefit versus Individual Patient Rights ........ 9
   - 3.3 Social Harm to Participants ........ 9
   - 3.4 Tensions between Data Protection and Innovation ........ 9
   - 3.5 Conflicts between Individual and Societal Perspectives ........ 10
   - 3.6 Impact of Power Imbalances on Sustainability ........ 10
   - 3.7 Responsible Use of Secondary Data Use ........ 10
   - 3.8 Ethics Applications and Ethics Committee Opinions ........ 11
4. Legal Foundations ........ 12
   - 4.1 Legal Starting Point ........ 12
   - 4.2 Principle of Permission ........ 12
   - 4.3 of Legal Authorization ........ 13
   - 4.4 Consent and Self-Determination ........ 13
   - 4.5 Societal Relevance of Research ........ 14
   - 4.6 Conclusion ........ 14

References ........ 15

# 1. Introduction

Health services research involves the use of a wide range of health data. Health data refers to information about patients that is collected during the routine provision of healthcare services (Cakir et al., 2025). Such data may include diagnoses, treatments, medications, or laboratory results, and are typically stored in administrative databases or electronic health records (Cakir et al., 2025). The term “health data” is deliberately used instead of “real-world data” or “real-world evidence” to emphasize that high-quality research can be grounded in data generated during routine care (Klinkhammer et al., 2020). Definitions provided by the Institute for Quality and Efficiency in Health Care (IQWiG) and the German Network for Health Services Research (DNVF) are also relevant in this context (Institut für Qualität und Wirtschaftlichkeit im Gesundheitswesen, 2020; Schmitt et al., 2023).

Health data serve as a foundation for numerous applications in both research and healthcare delivery, including support for decision-making and use in scientific investigations. They may take the form of registry data, routine data from statutory health insurance (GKV), or administrative data, and can be analyzed as either primary or secondary data (Schmitt et al., 2023).

The complexity of health data continues to grow in the context of increasing digitalization. It is therefore essential for professionals in both healthcare practice and research to develop a fundamental understanding of health data, particularly with regard to the associated ethical, legal, and social dimensions. This includes, for example, knowledge about informed consent procedures and the ability to recognize when legal review of data use is required (see Section 3 for details). Only with this awareness can responsibilities in handling health data be properly assessed and implemented. These considerations are relevant at all stages of the data life cycle—including collection, analysis, and reuse. It is therefore crucial that all individuals involved possess the competencies to reflect on their own actions, understand ethical, legal, and social challenges related to the use of health data, and make informed, autonomous decisions.

A foundational step in developing these competencies is an awareness of what constitutes science, what health data are, and how such data can be used for research purposes. Only with this understanding can professionals fully grasp the responsibility that comes with handling data—for instance, recognizing the difference between everyday observations and systematic investigation. This awareness is essential for understanding why scientific studies are subject to falsifiability. No single study can provide absolute certainty; metaphorically speaking, even if all observed swans are white, the existence of a single black swan proves the statement “All swans are white” to be false (Joas & Knöbl, 2020).

What does this imply for health data? A single study can never produce a universally valid conclusion. This highlights the importance of systematic reviews in medicine, which synthesize multiple findings. However, such scientific reasoning is also relevant in everyday life. The COVID-19 pandemic demonstrated that many actors in society struggled to understand why scientific insights and regulations changed frequently. It is not only scientists who need to be aware of this process, especially in light of the growing importance of science communication, but also healthcare professionals, who must be

able to interpret new regulations and explain them to patients, particularly in exceptional situations such as a pandemic. Understanding how scientific knowledge is produced is essential for interpreting institutional processes, structures, and decision-making. Moreover, researchers and medical professionals must be aware of their own roles in the collection, analysis, and dissemination of data in order to act responsibly.

The present ELSI concept (Ethical, Legal, and Social Implications) was developed within the project DIM.RUHR (Data Literacy Competence Center for the Interprofessional Use of Health Data in the Ruhr Metropolis). It outlines how the OER developed within the project can be meaningfully supplemented with foundational content related to data literacy in healthcare. This document is intended as a supporting resource. While it does not claim to be exhaustive, it aims to raise awareness of the complex interrelations involved in working with health data. Due to the multifaceted nature of the topic, the document highlights potential areas for action but focuses primarily on the key challenges and opportunities associated with the use of health data.

The ELSI concept is structured into three main areas: ethics in the use of health data, social aspects, and legal foundations.

# 2. Ethics in the Use of Health Data

The use of health data in healthcare involves a wide range of ethical considerations. These data are not only particularly sensitive but also critical for research, patient care, and public health policy. Their use requires continuous ethical reflection, especially in light of rapidly evolving technological and societal developments. This is particularly true for the use of artificial intelligence and automated decision-making systems in healthcare. As a result, ethical responsibility lies with all stakeholders, including those in research, clinical practice, policy, and education. A responsible approach to health data requires a basic understanding of key principles in medical ethics as well as methodological tools for ethical reflection. The following section introduces selected principles and presents a model for ethical decision-making in the context of health data use.

Beyond the points outlined here, established ethical standards and guidelines, such as professional codes and declarations (e.g. the Declaration of Helsinki in medical research), must be taken into account. Regulatory frameworks, including national and international health data legislation, anti-discrimination laws in healthcare, and liability rules, also shape the ethical landscape of health data use. Various methods are available for systematically evaluating ethical issues related to health data. Among them, the following are particularly relevant:

## 2.1 Principlism by Beauchamp and Childress

Ethical reflection in medicine often draws on the principlism model developed by Tom L. Beauchamp and James F. Childress. This model was strongly influenced by the Belmont Report (1978), which was drafted in response to the Tuskegee Syphilis Study (Rauprich, 2005). Shortly thereafter, Beauchamp and Childress published Principles of Biomedical Ethics (1979), which remains a foundational work in bioethics (Beauchamp and Childress, 2019). They argue that moral reasoning takes place on multiple levels: specific judgments are based on moral rules, which in turn are grounded in ethical principles, ultimately derived from ethical theories. Their model rests on the idea of "common morality", a shared sense of moral understanding that is presumed to be universally accessible. From this, they derive four central bioethical principles: respect for autonomy, non-maleficence, beneficence, and justice. These principles serve as a conceptual framework for discussing ethical issues across diverse contexts.

### 2.1.1 Respect for Autonomy

According to Beauchamp and Childress, this principle requires respect for individuals' autonomous decisions, as well as support for building or maintaining their capacity for autonomous decision-making (Beauchamp and Childress, 2019). In the context of health data, this means individuals have the right to make informed choices about how their data are used. The concept of data sovereignty aims to ensure not only that individuals are informed but that they actively participate in decisions about their data. The principle of autonomy thus underpins informed consent, which requires that individuals voluntarily, competently, and knowledgeably agree to medical interventions or data use. Ensuring transparency in how data are used is essential. Importantly, consent should not be seen as a one-time event

but must be revisited, especially as data use policies evolve. One major challenge is that many patients lack the technical background to fully understand the long-term consequences of data use. Clear communication and science communication are therefore essential.

### 2.1.2 Non-maleficence

Non-maleficence represents a negative obligation and, according to Beauchamp and Childress, is considered impartial (Beauchamp and Childress, 2019). While doing good for everyone may not always be possible, it is obligatory to avoid causing harm. With respect to health data, this means that no harm should result from their use, either for individuals or groups. This includes mitigating data protection risks and preventing misuse of sensitive information (e.g. by insurance companies or employers). Stakeholders must ensure that health data are protected through appropriate technical and organizational measures.

### 2.1.3 Beneficence

This principle obliges healthcare professionals and researchers to actively promote the well-being of others. In contrast to non-maleficence, beneficence entails a positive obligation to help and support (Beauchamp and Childress, 2019). Difficult ethical decisions may arise when the potential benefit to a group must be weighed against potential disadvantages for individuals. The use of health data should contribute to improving prevention, diagnosis, and treatment. However, this raises tensions between individual data protection and collective benefit. A balanced approach is needed to uphold individual rights while also realizing the broader potential of health data to support more effective research and care.

### 2.1.4 Justice

The principle of justice involves the fair distribution of resources, rights, and responsibilities. Various models of justice (distributive, procedural, and compensatory) guide decisions about equity. Since resources in healthcare are often limited, moral reasoning must be used to justify fair allocation. For example, equality may require giving everyone the same access to services, while equity may justify differential treatment, such as prioritizing patients in organ allocation.

In the context of health data, access to care and research must be fair and non-discriminatory. This includes reducing structural, technical, and linguistic barriers. Health data must be collected and processed in ways that are sensitive to justice concerns. Disproportionate representation of certain groups can introduce bias, leading to inequalities in both research and care. A key concern is ensuring equitable representation of all population groups in health data to avoid reinforcing existing disparities.

## 2.2 Balancing of Principles

The principles described above allow for ethical trade-offs and prioritization, depending on the context. This kind of balancing already plays a role in clinical settings, for example in ethics consultations.

## 2.3 Technology Assessmen

Another key area is technology assessment, which systematically examines the social, ethical, and ecological implications of new technologies. Various approaches exist to structure such assessments (Thokala and Duenas, 2012). In healthcare and biomedical research, technology assessment is essential for evaluating the long-term consequences of data-driven innovations. Beyond evaluating risks and opportunities, ethical guidelines for the use of new technologies must be developed. Participatory approaches are central to this effort. The development of ethical standards should be interdisciplinary and include all relevant actors, from researchers and medical professionals to policy makers.

## 2.4 Conclusion

An ethically informed approach to health data is essential for protecting patients' rights while advancing healthcare systems. The four principles of medical ethics (respect for autonomy, non-maleficence, beneficence, and justice) offer an initial point of orientation. However, complex decision-making processes require additional reflective methods to support sound judgments in practice. Moreover, modern technologies must be subject to ongoing ethical review to ensure they remain responsive to evolving societal and technological challenges. The following action points can be derived from these reflections:

- Ethical questions must be evaluated continuously and systematically, especially in the use of artificial intelligence and automated decision-making in healthcare. Societal and technological changes must be taken into account.
- Individuals must be informed clearly and transparently about how their data are used. Consent must be voluntary, informed, and competent, and must be revisited as data policies change. Personal data autonomy is central.
- Harm to individuals or groups must be avoided in the use of health data. Data protection risks and potential misuse must be actively mitigated. Technical and organizational safeguards are mandatory.
- Health data should actively contribute to improvements in prevention, diagnosis, and therapy. A balanced relationship between individual data protection, innovation, and societal benefit must be maintained.
- Access to healthcare and research must be fair and non-discriminatory. All population groups should be appropriately represented in health data to prevent bias and inequality.
- Ethical guidelines must be developed through interdisciplinary, participatory processes that involve all relevant stakeholders in research, clinical practice, and policy. Technology assessment is essential to establishing and evaluating ethical standards for new technologies in health research.

## 3. Social Aspects

Social aspects arise from societal norms and values that shape actions and interactions (for further explanation, see the Coleman bathtub model). They reflect the broader societal challenges and questions associated with the collection, analysis, and use of health data. Many of these issues have already been explored in disciplines such as sociology and political science. The corresponding literature provides a solid foundation for investigating ethical and societal questions in the context of health data (EUPATI).

A central issue concerns health inequalities, which are closely tied to social disparities such as socioeconomic status or social background. The hierarchical model proposed by Elkeles and Mielck (1997) explains the relationship between social and health inequalities by identifying several mediating factors. According to this model, social inequalities influence health inequalities primarily through the following mechanisms:

- **Accessibility of healthcare**
- **Exposure to health-related burdens**
- **Individual coping capabilities**

These factors impact health inequalities both directly and indirectly, for example through health-related behaviors (Elkeles and Mielck, 1997; Hurrelmann and Richter, 2013). Social inequalities are typically measured using indicators such as financial resources, education, and occupational or social prestige.

Another important concept is intersectionality, which refers to the simultaneous and overlapping presence of different forms of discrimination (Acker, 2000; Acker, 2010). Key dimensions include gender, ethnicity, and social class. In this context:

- Not only the data themselves can reflect bias (e.g. the gender health gap), but also the conclusions and analyses derived from them.
- Bias in data and among researchers can lead to distortion.
- There is a risk of social and societal harm to study participants.

This leads to the following action points:

- Health data must be collected, analyzed, and used in a way that prevents discrimination based on social origin, socioeconomic status, disability, or other factors.
- All population groups must have equal opportunities to participate in research and must not be disadvantaged through exclusion, particularly in clinical trials. Inclusion criteria should be designed to prevent the systematic exclusion of vulnerable individuals or groups based on gender or other characteristics.

- Case selection in research should be representative. This applies especially to studies based on random sampling (see Schnell et al., 2013), and marginalized groups should be intentionally included.
- Social, linguistic, or structural barriers that prevent participation in studies must be actively addressed through inclusive research practices.
- Health data must not be interpreted with biased assumptions about the health literacy of specific groups. Researchers and healthcare professionals need to be sensitized early to potential sources of bias.
- Social factors must be appropriately considered in the analysis of health data to mitigate health inequalities. Health data should be purposefully used to improve care for disadvantaged populations.
- Existing prejudices and stereotypes must not be reinforced through the analysis and use of health data. Comprehensive awareness of potential biases in research and care is necessary to avoid perpetuating structural inequality.
- Patients should be actively involved in decisions regarding the use of their health data. Patient-centered heuristics should be systematically integrated into research and care processes.
- Algorithms and bias can exacerbate health inequalities and result in discriminatory effects (e.g. in women's health, cancer research, or skin tone detection). Such algorithms and AI systems must be reviewed and, if necessary, adapted to ensure transparent and non-discriminatory decision-making in data-driven applications.
- People with disabilities and chronic conditions must be adequately represented in health data and research. A lack of representation must not lead to inferior healthcare or research outcomes.

Another key element of the social dimension is the power dynamics between actors, society, and the environment, particularly in the context of sustainability. According to Max Weber, power is defined as "the probability that one actor within a social relationship will be in a position to carry out their own will despite resistance" (Weber, 1976, p. 28). Such power imbalances often emerge between researchers and participants, creating ethical tensions. In studies on sensitive topics such as new therapies or medications, researchers must be especially mindful of these imbalances and work to mitigate them.

Interventions in research extend beyond medical procedures to include the observation of behavior and participation in experiments. In qualitative studies, such as those conducted in Facebook groups or online forums, the failure to disclose one's role as a researcher can present a significant ethical challenge. This is particularly true for private online groups, which are typically accessible only by invitation or approval. These groups demand heightened ethical scrutiny due to participants' increased expectations of privacy and confidentiality.

The central question in this context is: How can ethically responsible access to such communication spaces be designed without compromising the integrity of the research or the rights of those involved? Qualitative research in digital environments requires a high degree of contextual sensitivity and ethical

reflection. Access is often restricted not only by technical barriers but also by concerns related to transparency, informed consent, and trust within the studied communities. From an ethical standpoint, openly disclosing one's role as a researcher is generally considered best practice, aligning with the principle of informed consent. However, in practical terms, such transparency may lead to exclusion from the group or altered communication dynamics, including behavioral changes among members, which can bias the data. In contrast, covert access may offer more authentic insights into informal communication patterns but presents a significant ethical dilemma. Intentionally concealing the researcher's identity can be considered deceptive and contradicts the ethical guidelines of many academic institutions and professional organizations.

For this reason, researchers must carefully weigh methodological strategies in advance. Ideally, access strategies should be reviewed by an ethics committee and clearly justified in the research design. Alternative approaches might include negotiating observer status with group moderators, ensuring strict anonymization of sensitive data, or focusing on public forums with comparable structures. Unlike private groups, public platforms such as Instagram or X (formerly Twitter) operate in an open-access format. Content and interactions, posts, comments, reactions, are typically intended for a wide audience and are readily accessible.

From a methodological perspective, this significantly changes the nature of data collection and analysis. Gathering publicly available data through methods such as web scraping is technically straightforward and ethically less problematic, since the information was not shared in a private context. It is generally assumed that users are aware of the visibility of their posts, particularly if they have not adjusted their privacy settings. This distinction is critical not only for choosing appropriate research methods but also for maintaining a clear ethical stance in digital research environments. In the age of big data, vast amounts of information can be collected and analyzed, not only by researchers, but also by corporations, institutions, and public organizations. However, the availability of such data also increases the potential for misuse.

A historical example of extreme data misuse is the secondary use of census data during the Third Reich to facilitate the genocide of Jews, Roma, and others (Seltzer and Anderson, 2008). Today, it remains difficult or even impossible to predict who may gain access to collected data in the future. This raises fundamental questions regarding data security and participant protection. Social inequalities may also emerge in relation to access to new medications: who receives them, how they are financed, and who has access to the underlying data. These are all critical societal questions that must be addressed (see also inequality through intersectionality). Moreover, existing power structures can lead to conflicts of interest. Researchers may find themselves caught between scientific curiosity, economic incentives, and ethical responsibility. Goffman's role theory can help analyze how actors perform their roles in different social contexts and how this influences power relations in research.

Power dynamics also affect the academic field itself. As Richard Münch has shown, research funding and publication output are influenced by institutional prestige. Institutions with prominent researchers or established projects are often granted more funding and recognition, leading to further advantages in terms of visibility and publication (Münch, 2017). This is relevant to the social dimension of data use

because important research topics may be overlooked or underfunded due to these structural dynamics, ultimately impacting society as a whole.

From these considerations, several implications arise that should guide the use and study of health data:

## 3.1 Power Imbalances between Researchers and Participants

Power differentials between researchers and study participants can substantially limit the latter's decision-making autonomy. Unequal relationships may lead participants to feel inadequately informed about potential risks or pressured to enroll in a study. Researchers should therefore provide transparent, comprehensible information, ensure thorough briefing, and actively involve participants in all decisions. Participatory research designs and independent ethics committees can further promote a fair and respectful research environment.

## 3.2 Scientific Benefit versus Individual Patient Rights

Researchers must reconcile scientific progress with the individual rights of patients. Safeguarding sensitive data and the right to informational self-determination must remain paramount. Placing research benefit above patient protection entails serious ethical and legal risks, including data misuse and insufficient disclosure of study participation. In narrowly defined public-health contexts, limited and strictly regulated data-protection exemptions may generate societal benefit, yet uneven academic power structures often determine which topics receive funding, thereby hindering social justice and equitable participation. Transparent decision procedures, independent oversight bodies, and stronger patient involvement are essential for upholding ethical standards.

## 3.3 Social Harm to Participants

Misuse of sensitive data can expose participants to discrimination, stigmatization, and social disadvantage. Uncontrolled access may amplify existing inequalities by subjecting certain groups to a higher risk of exclusion or unequal treatment. Researchers and institutions have a duty to minimize these risks through robust data-protection measures, transparent practices, and continual ethical reflection. Clear regulations, independent monitoring, and the active inclusion of affected groups are required to prevent harm and ensure just use of health data.

## 3.4 Tensions between Data Protection and Innovation

Strict data-protection regimes can hinder scientific innovation by limiting access to essential research data, yet ethical safeguards are indispensable for preventing misuse. A workable balance between individual data security and collective benefit can be achieved through transparent data-protection policies, anonymized processing, clear consent procedures, and the involvement of independent ethics committees.

### 3.5 Conflicts between Individual and Societal Perspectives

Research must consider individual interests without obstructing societal progress. Conflicts arise when personal data-protection rights collide with potential public benefit, for example in disease prevention. Although findings often serve the broader population, some groups may benefit disproportionately or suffer disadvantages due to unequal representation. Fair distribution of benefits and burdens requires transparent decisions, inclusive research designs, and safeguards for vulnerable populations. Therefore, an ethically grounded balance between personal privacy and societal gain is indispensable.

### 3.6 Impact of Power Imbalances on Sustainability

Power relations within research can strongly influence the implementation of sustainability goals. Economic and scientific interests may conflict with ecological and social priorities, delaying or neglecting sustainable solutions. Researchers should integrate environmental and sustainability considerations early, promote interdisciplinary approaches, and make transparent decisions that account for long-term ecological and social consequences rather than short-term economic gains. Fair resource allocation, ethical reflection, and participatory methods can help balance scientific progress with sustainable development.

### 3.7 Responsible Use of Secondary Data Use

The responsible handling of secondary data, particularly those originating from hospitals or similar institutions, requires careful balancing of the potential benefits for research with the need to protect the privacy of individuals. Secondary data that were originally collected for other purposes can provide valuable insights into health trends and treatment outcomes. However, researchers must ensure that such data are anonymized and securely stored to protect patient identities. Moreover, secondary data must be critically examined with respect to the previously discussed criteria, such as intersectionality and power relations, especially regarding potential biases. The fact that data are collected in the context of healthcare does not guarantee their objectivity or generalizability. For example, data from general practices in socioeconomically privileged areas may differ significantly from those collected in underserved regions, reflecting different healthcare needs and service patterns. These structural differences are often not documented in the available metadata and may not be transparently communicated by data providers. It is therefore essential to systematically identify and reflect on such biases during data analysis in order to avoid drawing misleading conclusions or reproducing social inequalities in research outcomes.

In addition, the use of such data must be transparent and traceable. Patients should be informed about the potential use of their data. Ethics committees play a central role in assessing the appropriateness of data use and ensuring compliance with applicable legal standards. Social responsibility also requires that research findings be used for the benefit of society and not lead to discrimination or stigmatization. From a social perspective, the same recommendations outlined above apply.

## 3.8 Ethics Applications and Ethics Committee Opinions

In medical research, obtaining an ethics opinion from a competent ethics committee is a central requirement for conducting empirical studies involving patients or research participants. This formal review process is designed to protect individuals from potential physical, psychological, or social risks and to ensure that research projects are conducted in accordance with applicable legal and ethical standards. The ethics application usually includes a detailed description of the study design, recruitment strategy, measures for ensuring data protection and confidentiality, and procedures for obtaining informed consent. The committee's opinion may result in approval, approval with conditions, or rejection. This assessment is legally binding, particularly in the context of clinical trials or studies involving vulnerable populations.

In addition to legal safeguards, the ethics application encourages researchers to reflect systematically on the ethical implications of their work. In interdisciplinary fields, such as those situated at the intersection of medicine, engineering, psychology, and the social sciences, ethical review is increasingly regarded as a marker of good scientific practice, even for non-interventional studies.

# 4. Legal Foundations

Legal regulation aims to ensure compliance with the ethical and social principles outlined above. In research contexts, various legal frameworks apply. As mentioned earlier, clinical research generally requires the approval of a competent ethics committee. In pharmaceutical research, for example, this requirement is based on Sections 41 and following of the German Medicinal Products Act (Arzneimittelgesetz, AMG). For the development of medical devices, the obligation arises from Regulation (EU) 2017/745 on medical devices (Medical Device Regulation, MDR) in conjunction with the German Medical Devices Implementation Act (MPDG). Additional relevant provisions can be found in the professional codes of conduct issued by the state chambers of physicians and other regulations.

Similarly, data use is context dependent and must be assessed in relation to specific legal requirements. For instance, ecclesiastical data protection law may apply if research is conducted in hospitals run by religious organizations. If routine data from social insurance carriers are used, the relevant provisions of social law come into play. Above these specific laws stands constitutional law, which provides uniform standards for the design of statutory regulations. A constitutional perspective helps to identify when more in-depth legal assessment is required.

## 4.1 Legal Starting Point

From a legal perspective, the key issue is the tension between data protection on the one hand and the public interest in scientific knowledge on the other. The starting point for regulation is the general right to personality, which the German Federal Constitutional Court (Bundesverfassungsgericht, BVerfG) derives from Article 2, paragraph 1 in conjunction with Article 1, paragraph 1 of the Basic Law (Grundgesetz, GG). One expression of this general right is the fundamental right to informational self-determination.

> **Note**: In its landmark decision on the census law in 1983, the BVerfG established the right to informational self-determination as part of the general personality right. The court held that "based on the principle of self-determination," individuals must retain "the authority to decide in principle on the disclosure and use of their personal data" (BVerfG, Judgment of December 15, 1983 – 1 BvR 209/83 et al., BVerfGE 65, 1 ff.).

This right implies that individuals should, in principle, have control over how their personal data are used. Consequently, a general prohibition with reservation of permission applies.

## 4.2 Principle of Permission

A prohibition with reservation of permission inverts the usual legal assumption: not everything is permitted unless expressly forbidden, but rather, everything is forbidden unless expressly permitted. Each specific use of data must be based on a legal provision that explicitly authorizes it.

Thus, the first legal step in handling data is to identify the legal basis on which the intended data use relies.

### 4.3 of Legal Authorization

The courts have established clear requirements for interference with the right to informational self-determination. In the context of health data, the principle of purpose limitation is particularly important. According to this principle, the law authorizing data collection must clearly define the permissible purposes for which the data may be used or further processed.

If legislators permit the use of existing data beyond the original purpose for which they were collected, they must establish a new legal basis for that extended use. Once the legal basis for data use is identified, it must be verified whether the intended use aligns with the original purpose.

> **Note**: Health data may have been collected under different legal frameworks. The legal basis for initial collection is not automatically valid for subsequent use. If data use exceeds the original purpose, the new use must be specifically authorized by law. For example, when using routine data from health insurance providers, special data protection regulations under social law must be observed.

After identifying a potential legal basis, it must be determined whether the intended data use is actually covered by that basis. Any interference with the right to informational self-determination must be justified, which includes assessing proportionality. This applies both to the creation of the legal basis and to its application in individual cases.

### 4.4 Consent and Self-Determination

Legal authorization provisions typically include requirements concerning the consent of data subjects. However, it is not excluded that data use may also be legally permitted without the individual's consent. Whether the legislature is permitted to enact such provisions depends significantly on the principle of proportionality. In simplified terms, any legislative provision must pursue a legitimate purpose. It must be suitable for achieving that purpose, necessary, and ultimately proportionate in the narrower sense. At its core, this involves evaluating the relation between the intended purpose and the means used. In this case, the restriction of the right to informational self-determination is weighed against the purpose of data collection.

> **Note:** In this context, requirements for anonymization and pseudonymization become particularly relevant. The infringement on informational self-determination is generally less severe when the personal reference of the data has been removed. From a legal-theoretical perspective, there is no infringement of the right to informational self-determination if genuine anonymization has been achieved and the data can no longer be linked to individuals. In practice, however, it is highly contested when anonymization is considered fully effective. In cases of

doubt, legal practice tends to assume that an infringement has occurred. Therefore, anonymization and pseudonymization are especially relevant in assessing the intensity of the interference.

According to established case law (see in detail Huber/Voßkuhle/Eichberger, GG Art. 2, para. 298 with further references), it is well established that data collection for unspecified future purposes is inadmissible. The purpose of data collection must be sufficiently defined. For example, the creation of a general data repository for unspecified health research purposes would be unconstitutional. This requirement interacts with the standards for obtaining consent: briefly put, even a significant interference with the right to informational self-determination may be based on a broadly worded legal provision — but this requires a correspondingly precise consent declaration. If the legal provision does not clearly define the purpose of the data collection, then the content of the consent must be all the more specific. Ultimately, what counts is the outcome: the legal framework must ensure the ethical use of data (see also Section 2).

### 4.5 Societal Relevance of Research

Another factor relevant to assessing proportionality is the societal importance of the research. An infringement of the right to informational self-determination is more likely to be justified when a socially significant gain in knowledge is expected. What is crucial is that the research project complies with the principles of good scientific practice and incorporates relevant ethical and social considerations.

### 4.6 Conclusion

Requirements for handling health data are complex in many respects. As a result, the current legal framework is subject to fundamental criticism, and the design of legal regulation, particularly data protection requirements, is often viewed as a barrier to medical progress (see Bundestag printed paper 20/9046, p. 1).

Nevertheless, it should be emphasized that in the preparatory phase of data collection, extensive opportunities exist to shape data protection through informed consent and contractual agreements. Legal obstacles can generally be addressed through anticipatory planning. This summary of legal principles is therefore intended to raise awareness of potential pitfalls in working with health data and to promote legally and ethically responsible data practices.

## References


Acker, J. (2000). Revisiting class: Thinking from gender, race, and organizations. *Social Politics: International Studies in Gender, State & Society, 7*(2), 192–214. https://doi.org/10.1093/sp/7.2.192

Acker, J. (2010). Geschlecht, Rasse und Klasse in Organisationen – die Untersuchung von Ungleichheit aus der Perspektive der Intersektionalität. *Feministische Studien, 28*(1). https://doi.org/10.1515/fs-2010-0109

Beauchamp, T. L., & Childress, J. F. (2019). *Principles of biomedical ethics* (8th ed.). Oxford University Press.

Cakir, M., Starke, P., Nolting, A., Qu, W., Pieper, D., & Mathes, T. (2025). Versorgungsnahe Daten zur Bewertung der vergleichenden Effektivität von medizinischen Behandlungen: eine Bestandsaufnahme der verfügbaren Datenquellen in Deutschland unter besonderer Berücksichtigung von Registern. *Zeitschrift für Evidenz, Fortbildung und Qualität im Gesundheitswesen, 194*, 1–7. https://doi.org/10.1016/j.zefq.2025.01.008

Elkeles, T., & Mielck, A. (1993). *Soziale und gesundheitliche Ungleichheit. Theoretische Ansätze zur Erklärung von sozioökonomischen Unterschieden in Morbidität und Mortalität*. WZB Wissenschaftszentrum Berlin für Sozialforschung.

Elkeles, T., & Mielck, A. (1997). Ansätze zur Erklärung und Verringerung gesundheitlicher Ungleichheit. *Jahrbuch für Kritische Medizin, 26*, 23–44.

Huber, P., & Voßkuhle, A. (2024). *Grundgesetz: GG* (8. Aufl.).

Hurrelmann, K., & Richter, M. (2013). *Gesundheits- und Medizinsoziologie. Eine Einführung in sozialwissenschaftliche Gesundheitsforschung* (8., überarb. Aufl.). Juventa Verlag.

Institut für Qualität und Wirtschaftlichkeit im Gesundheitswesen. (2020). *Konzepte zur Generierung versorgungsnaher Daten und deren Auswertung zum Zwecke der Nutzenbewertung von Arzneimitteln nach § 35a SGB V* (Version 1.1).

Klinkhammer-Schalke, M., Kaiser, T., Apfelbacher, C., Benz, S., Dreinhöfer, K. E., Geraedts, M., Hauptmann, M., Hoffmann, F., Hoffmann, W., Koller, M., Kostuj, T., Kowalski, C., Mugele, K., Ortmann, O., Schmitt, J., Schünemann, H., Veit, C., Wesselmann, S., & Bierbaum, T. (2020). Manual für Methoden und Nutzung versorgungsnaher Daten zur Wissensgenerierung. *Gesundheitswesen, 82*(8–09), 716–722. https://doi.org/10.1055/a-1237-4011

Joas, H., & Knöbl, W. (2020). *Sozialtheorie. Zwanzig einführende Vorlesungen* (6. Aufl., aktualisierte, mit einem neuen Vorwort versehene Ausgabe). Suhrkamp.

Münch, R. (2007). *Die akademische Elite. Zur sozialen Konstruktion wissenschaftlicher Exzellenz*. Suhrkamp.

Rauprich, O. (2005). Prinzipienethik in der Biomedizin – Zur Einführung. In O. Rauprich & F. Steger (Hrsg.), *Prinzipienethik in der Biomedizin. Moralphilosophie und medizinische Praxis*. Campus Verlag GmbH.

Schnell, R., Esser, E., & Hill, P. B. (2013). *Methoden der empirischen Sozialforschung* (10., überarb. Aufl.). Oldenbourg.

Schmitt, J., Bierbaum, T., Geraedts, M., Gothe, H., Härter, M., Hoffmann, F., Ihle, P., Kramer, U., Klinkhammer-Schalke, M., Kuske, S., March, S., Reese, J.-P., Schoffer, O., Swart, E., Vollmar, H. C., Walther, F., & Hoffmann, W. (2023). Das Gesundheitsdatennutzungsgesetz – Potenzial für eine bessere Forschung und Gesundheitsversorgung. *Gesundheitswesen*. https://doi.org/10.1055/a-2050-0429

Thokala, P., & Duenas, A. (2012). Multiple criteria decision analysis for health technology assessment. *Value in Health, 15*(3), 1172–1181. https://doi.org/10.1016/j.jval.2012.06.015

Weber, M. (1976). *Wirtschaft und Gesellschaft. Grundriss der verstehenden Soziologie* (5., rev. Aufl.). Mohr.
